# Supplementary figures and images for: Mouse dead end1 acts with Nanos2 and Nanos3 to regulate testicular teratoma incidence
Source: PLoS One. 2020 Apr 27;15(4):e0232047. doi: 10.1371/journal.pone.0232047 (PMC7185693; doi:10.1371/journal.pone.0232047)

Figure S1. Imai A, et al.

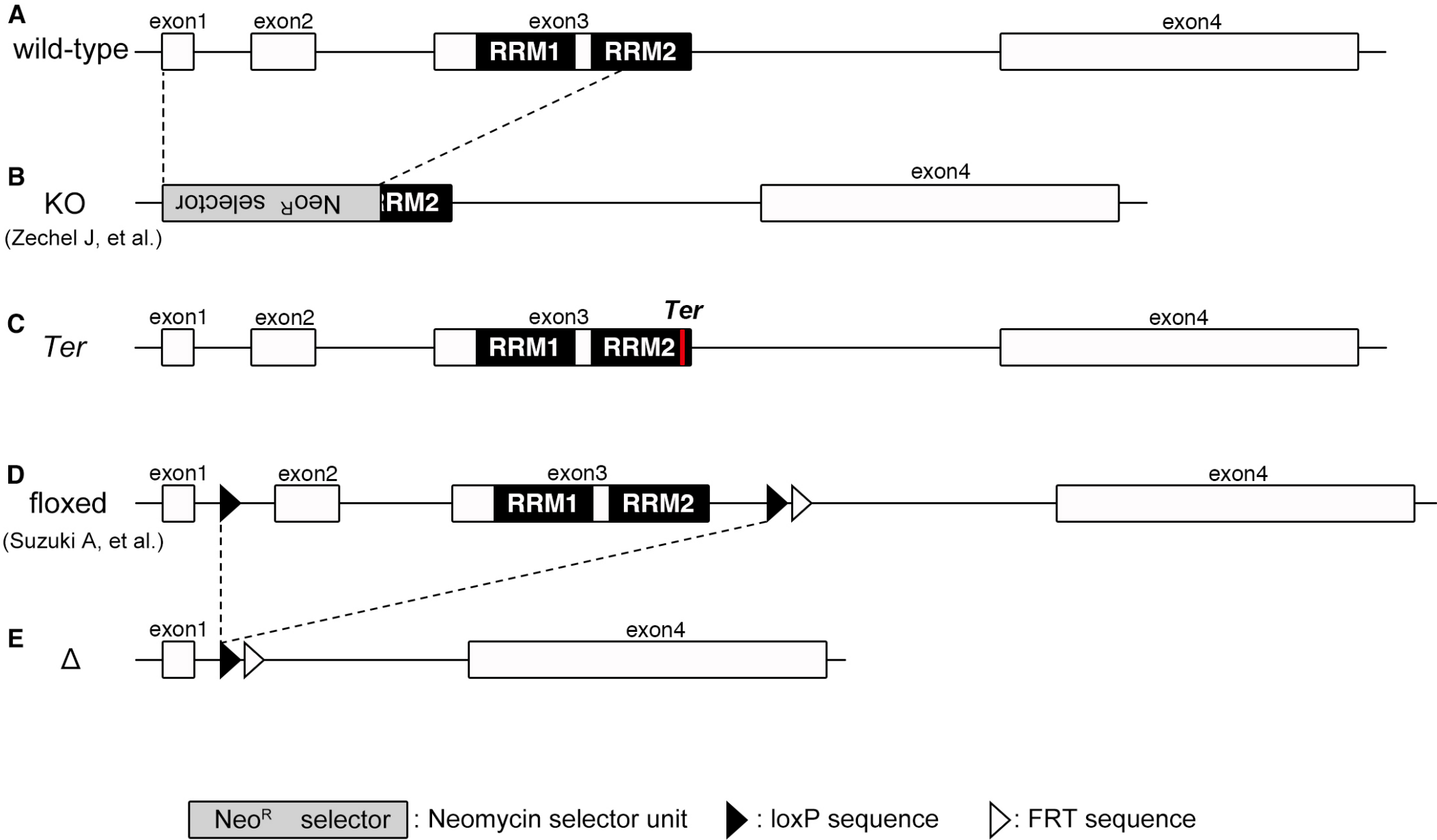

Supplement: S1 Fig — Structures of the Dnd1 (A-E) wild-type allele (A), KO allele generated by Zechel et al. [16] (B), Ter allele [9] (C), floxed allele generated by Suzuki et al. [14] (D), and Δ allele (E). (PDF) [file pone.0232047.s001.pdf]

Figure S2. Imai A, et al.

129-4W testes

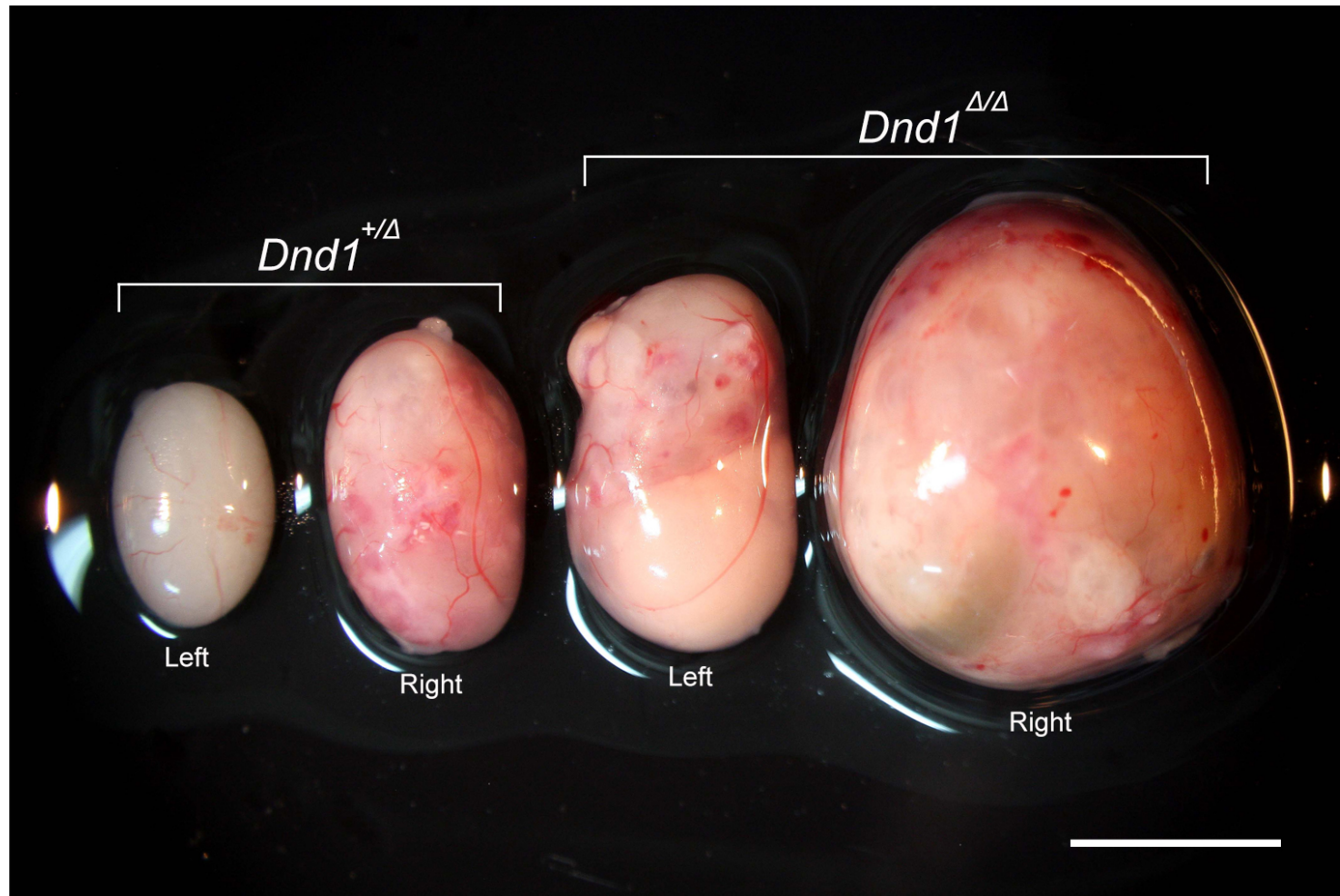

Supplement: S2 Fig — Comparison of the testes from 4-week-old Dnd1+/Δ and Dnd1Δ/Δ mice of the 129 strain. Note that testicular teratomas developed only in the right testis in the Dnd1+/Δ mice, whereas both testes have testicular teratoma in the Dnd1Δ/Δ mice. Scale bar: 5 mm. (PDF) [file pone.0232047.s002.pdf]

Figure S3. Imai A, et al.

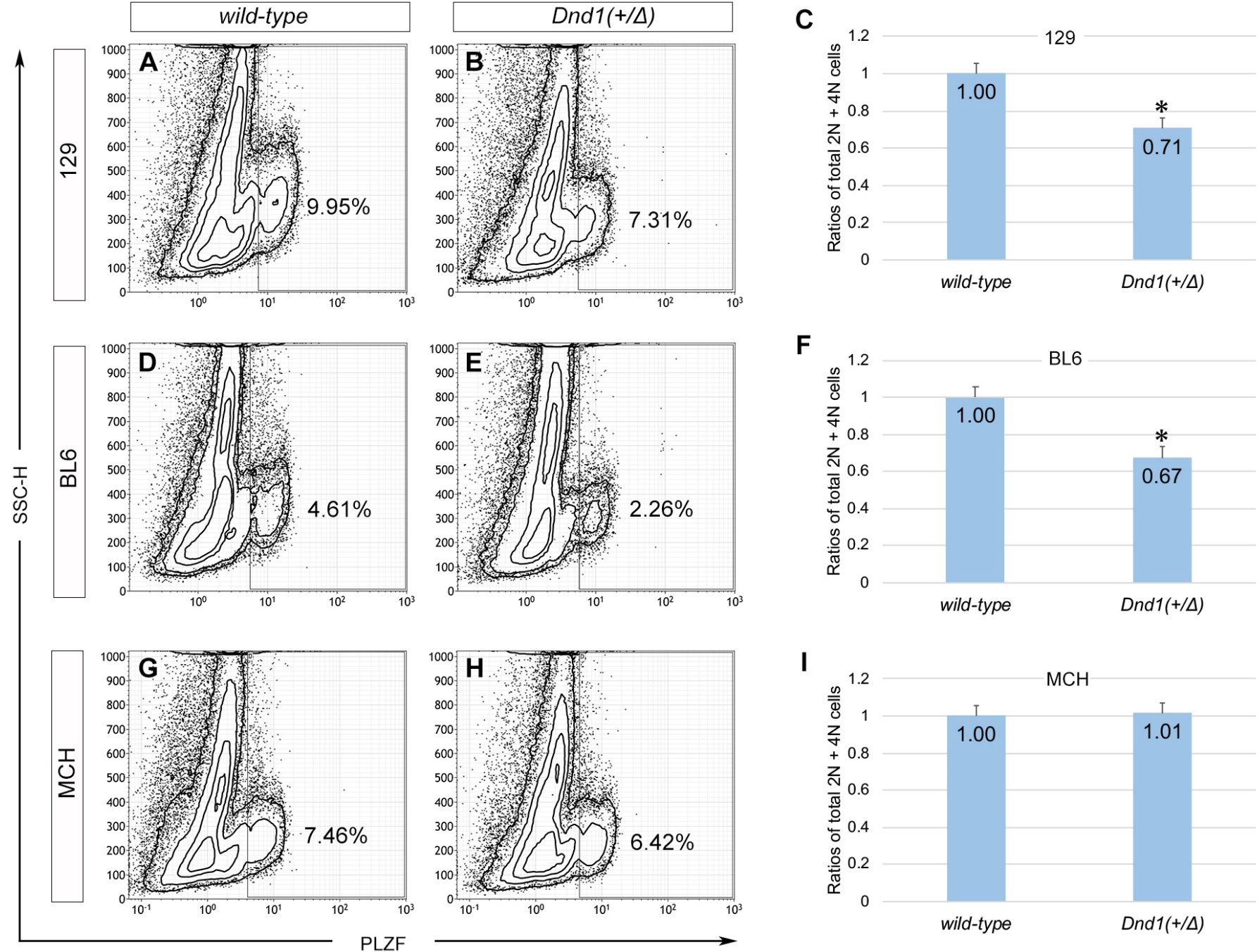

Supplement: S3 Fig — Representative flow cytometric analyses of testis cells from 12-week-old wild-type (A, D, G) and Dnd1+/Δ (B, E, H) mice of the 129 (A–C), BL6 (D–F), and MCH (G–I) strains for PLZF. Percentages of cells within each PLZF-positive gate (A, B, D, E, F, G) were normalized by the ratio of PLZF-positive cells from wild-type mice and indicated (C, F, I). Error bars represent mean ± SD; three mice were analyzed per genotype and strain. *P < 0.05 (Student’s t-test). (PDF) [file pone.0232047.s003.pdf]

Figure S4. Imai A, et al.

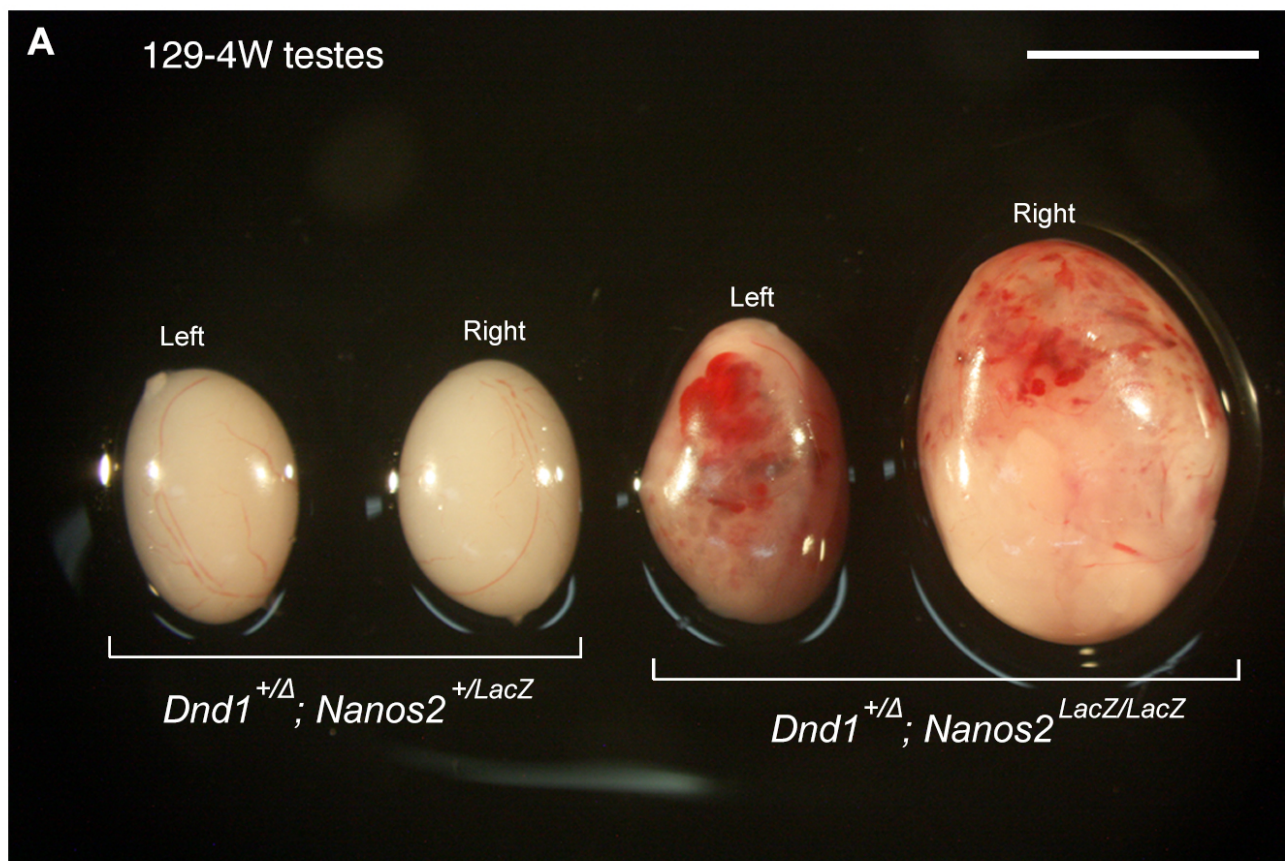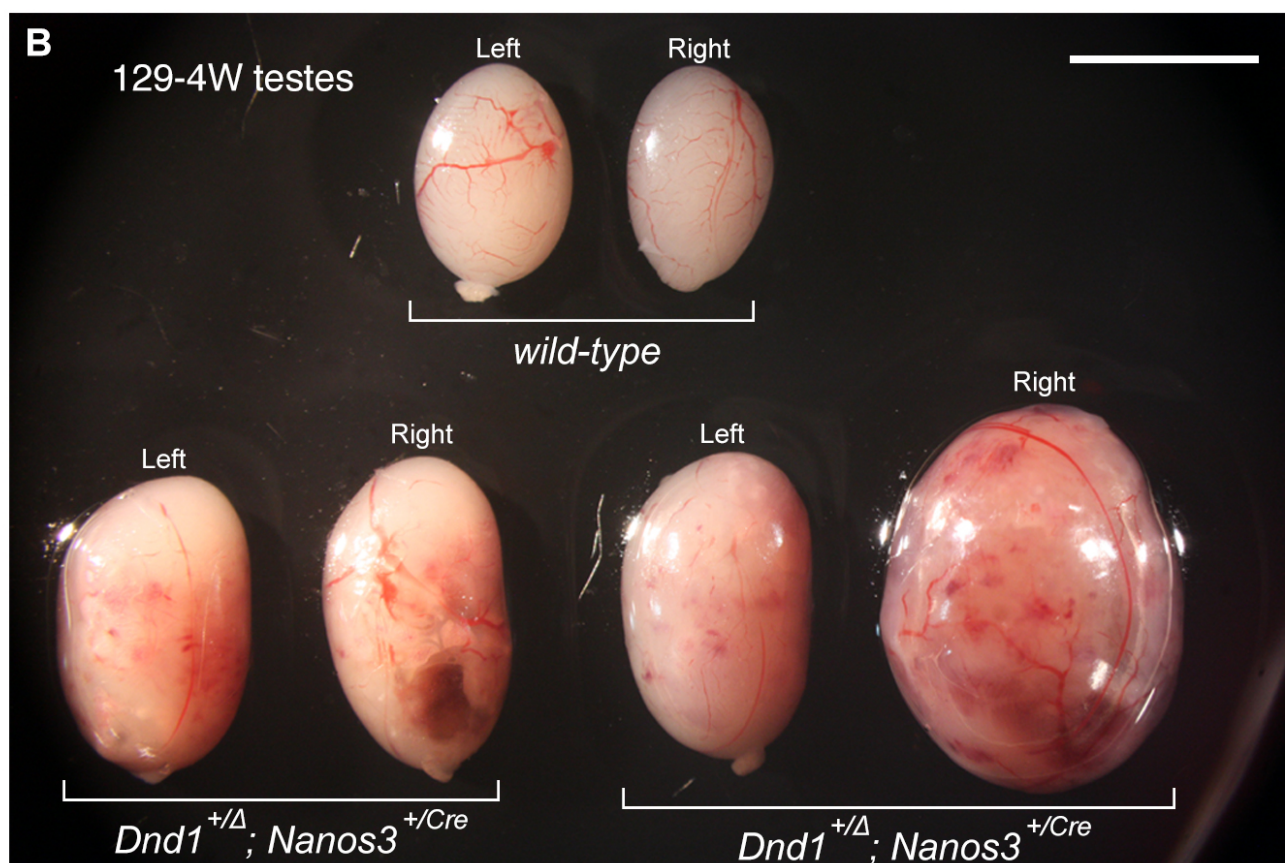

Supplement: S4 Fig — Comparison of the testes from 4-week-old Dnd1+/Δ; Nanos2+/LacZ and Dnd1+/Δ; Nanos2LacZ/LacZ mice (A), or wild-type and Dnd1+/Δ; Nanos3+/Cre mice of the 129 strain (B). Scale bars: 5 mm in (A) and (B). (PDF) [file pone.0232047.s004.pdf]

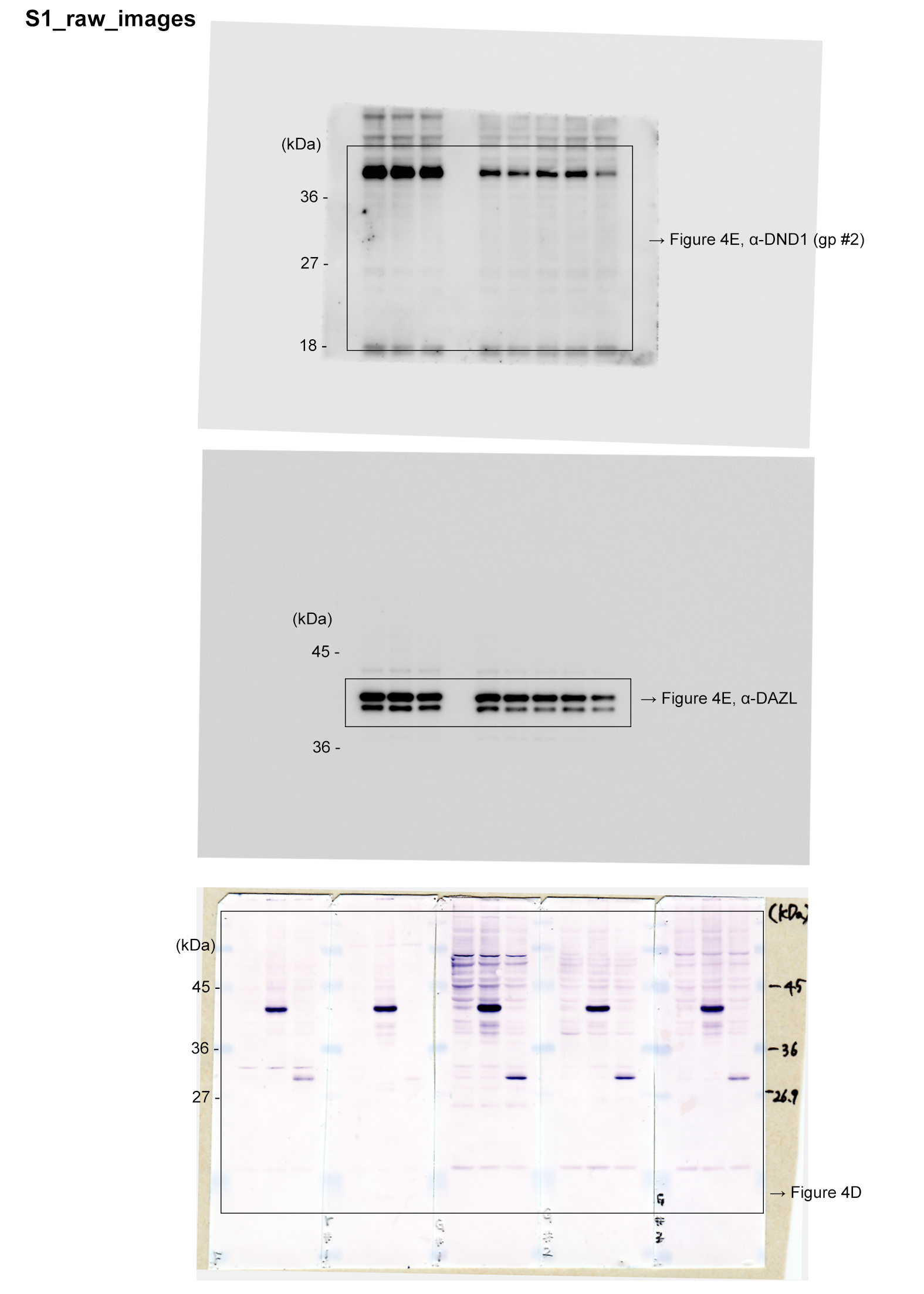

Supplement: S1 Raw images — (JPG) [file pone.0232047.s006.jpg]
