# Supplementary material for: Mouse dead end1 acts with Nanos2 and Nanos3 to regulate testicular teratoma incidence
Source: PLoS One. 2020 Apr 27;15(4):e0232047. doi: 10.1371/journal.pone.0232047 (PMC7185693; doi:10.1371/journal.pone.0232047)
Supplement: S5 Fig — The 15 genes neighboring Dnd1 are listed in order of their location on chromosome 18. Their knockout mouse (KO) lines and phenotypes are also mentioned in the list. IMPC: The International Mouse Phenotyping Consortium. (PDF) [file pone.0232047.s005.pdf]

**Figure S5. Imai A, et al.**

| | gene | location on Ch. 18 | konckout (KO) mouse line | phenotype in KO mouse |
|---|---|---|---|---|
| 1 | *Vaultrc5* | 36,801,763..36,802,107 | not reported | |
| 2 | *Zmat2* | 36,793,923..36,799,660 | not reported | |
| 3 | *Hars2* | 36,783,202..36,792,562 | generated by IMPC | Phenotyping has not been finished |
| 4 | *Hars* | 36,766,528..36,783,205 | not reported | |
| 5 | *Dnd1* | 36,763,671..36,766,214 | | |
| 6 | *Wdr55* | 36,760,222..36,763,708 | Yougren KK, et al. *Nature* . 2005 | Embryonic lethal |
| 7 | *Ik* | 36,744,656..36,757,639 | generated by IMPC | Embryonic lethal , Preweaning lethal |
| 8 | *Ndufa2* | 36,742,332..36,744,587 | not reported | |
| 9 | *Tmco6* | 36,735,019..36,742,400 | generated by IMPC | Preweaning lethal, Increased or absent threshold for auditory brainstem response |
| 10 | *Cd14* | 36,725,064..36,726,815 | Haziot A, et al. *Immunity* . 1996 | Resistant to septic shock |
| 11 | *Gm41699* | 36,709,020..36,718,962 | not reported | |
| 12 | *E230025N22Rik* | 36,684,923..36,696,091 | not reported | |
| 13 | *Slc35a4* | 36,679,215..36,683,862 | not reported | |
| 14 | *Apbb3* | 36,671,154..36,679,366 | generated by IMPC | No significant phetypes reported |
| 15 | *Sra1* | 36,667,187..36,670,311 | Liu S, et al. *Journal of Biological Chemistry* . 2014 | Resistant to high fat diet obesity |
| 16 | *Eif4ebp3* | 36,664,060..36,666,324 | generated by IMPC | Increased vertical activity , Decreased monocyte cell number |
